# A multicenter double-blind randomized crossover study comparing the impact of dorsal subthalamic nucleus deep brain stimulation versus standard care on apathy in Parkinson’s disease: a study protocol

**DOI:** 10.1186/s13063-024-07938-9

**Published:** 2024-02-03

**Authors:** T. J. C. Zoon, G. van Rooijen, M. F. Contarino, S. van der Gaag, R. Zutt, J. T. van Asseldonk, P. van den Munckhof, P. R. Schuurman, D. A. J. P. Denys, R. M. A. de Bie

**Affiliations:** 1 Department of Psychiatry, Amsterdam UMC, Location AMC, Amsterdam, the Netherlands; 2 HagaZiekenhuis, the Hague, the Netherlands; 3 Elisabeth-TweeSteden Ziekenhuis, Tilburg, the Netherlands; 4 Department of Neurosurgery, Amsterdam UMC, Amsterdam, the Netherlands; 5 Department of Neurology, Amsterdam UMC, Location AMC, Amsterdam, the Netherlands

**Keywords:** Neurology, Parkinson’s disease, Apathy, Deep brain stimulation, Randomized controlled trial

## Abstract

**Background:**

Neuroimaging studies suggest an association between apathy after deep brain stimulation (DBS) and stimulation of the ventral part of the subthalamic nucleus (STN) due to the associative fibers connected to the non-motor limbic circuits that are involved in emotion regulation and motivation. We have previously described three patients with severe apathy that could be fully treated after switching stimulation from a ventral electrode contact point to a more dorsal contact point.

**Objectives:**

To determine whether more dorsal stimulation of the STN decreases apathy compared to standard care in a multicenter randomized controlled trial with a crossover design.

**Methods:**

We will include 26 patients with a Starkstein Apathy Scale (SAS) score of 14 or more after subthalamic nucleus (STN) deep brain stimulation (DBS) for refractory Parkinson’s disease. This is a multicenter trial conducted in two teaching hospitals and one university medical center in the Netherlands after at least 3 months of STN DBS. Our intervention will consist of 1 month of unilateral dorsal STN stimulation compared to treatment as usual. The primary outcome is a change in SAS score following 1 month of DBS on the original contact compared to the SAS score following 1 month of DBS on the more dorsal contact. Secondary outcomes are symptom changes on the Movement Disorders Society-Unified Parkinson’s Disease Rating Scale motor part III, Montgomery-Åsberg Depression Rating Scale, 39-item Parkinson’s disease questionnaire, Parkinson’s disease impulsive-compulsive disorders questionnaire, changes in levodopa-equivalent daily dosage, apathy rated by the caregiver, and burden and quality of life of the caregiver.

**Trial registration:**

ClinicalTrials.gov NL8279. Registered on January 10, 2020.

**Supplementary Information:**

The online version contains supplementary material available at 10.1186/s13063-024-07938-9.

## Introduction

### Background and rationale {6a}

Deep brain stimulation (DBS) of the subthalamic nucleus (STN) reduces off-related motor symptoms by up to 60% in refractory Parkinson’s disease (PD) [1, 2]. STN DBS also has a beneficial effect on many non-motor symptoms (i.e., depressive symptoms, anxiety, hyperdopaminergic disinhibited behaviors) [3]. Apathy, defined as the loss of motivation, is an important exception as it increases after STN DBS, with a prevalence of 21 to 78% [4, 5]. This increase of apathy is clinically relevant considering its negative effect on a patient’s quality of life and the drastic influence on the lives of caregivers [6–8]. Apathy is currently understood as a state of decreased motivation which is presented as decreased goal-directed behaviors with concurrent reduced interests and emotional flatness [9, 10]. Apathy is thought to be an independent syndrome caused by dysfunction of four different but interrelated cognitive domains: “emotional resonance reduction,” “depression,” “executive dysfunction dysfunction,” and “auto-activation deficiency” [9]. Neurobiologically, apathy is thought to be related to reward-related circuits in the orbitomedial and ventromedial prefrontal cortex, amygdala, and nucleus accumbens [9, 11, 12]. Several risk factors have been identified. First, dopaminergic medication is commonly reduced following STN DBS and is a likely contribution for the occurrence of apathy. Hence, apathy can also be treated with dopamine agonists, although this effect is indisputable [4, 6, 13, 14]. Second, apathy is associated with cognitive decline independent of STN DBS, which can be seen as disease progression and is not likely influenced by dopaminergic- or stimulation-based treatments [9, 15]. Lastly, neuroimaging studies suggest an association between apathy after DBS and stimulation of the ventral part of the STN due to the associative fibers connected to the non-motor limbic circuits that are involved in emotion regulation and motivation [16, 17]. We have previously described three patients with severe apathy that were treated by switching stimulation from a ventral electrode contact point to a more dorsal contact point [18].

### Objectives {7}

We will test whether STN DBS-related apathy could be treated by switching stimulation from a ventral to a more dorsal contact point on the electrode. Additionally, we aim to investigate whether these adjusted stimulation parameters have effects on PD motor manifestations, psychiatric symptoms, and quality of life of the participant and their caregivers. 

### Trial design {8}

This is a multicenter prospective randomized open-label blinded endpoint crossover superiority trial.

## Methods: participants, interventions, and outcomes

### Study setting {9}

**Fig. 1:**
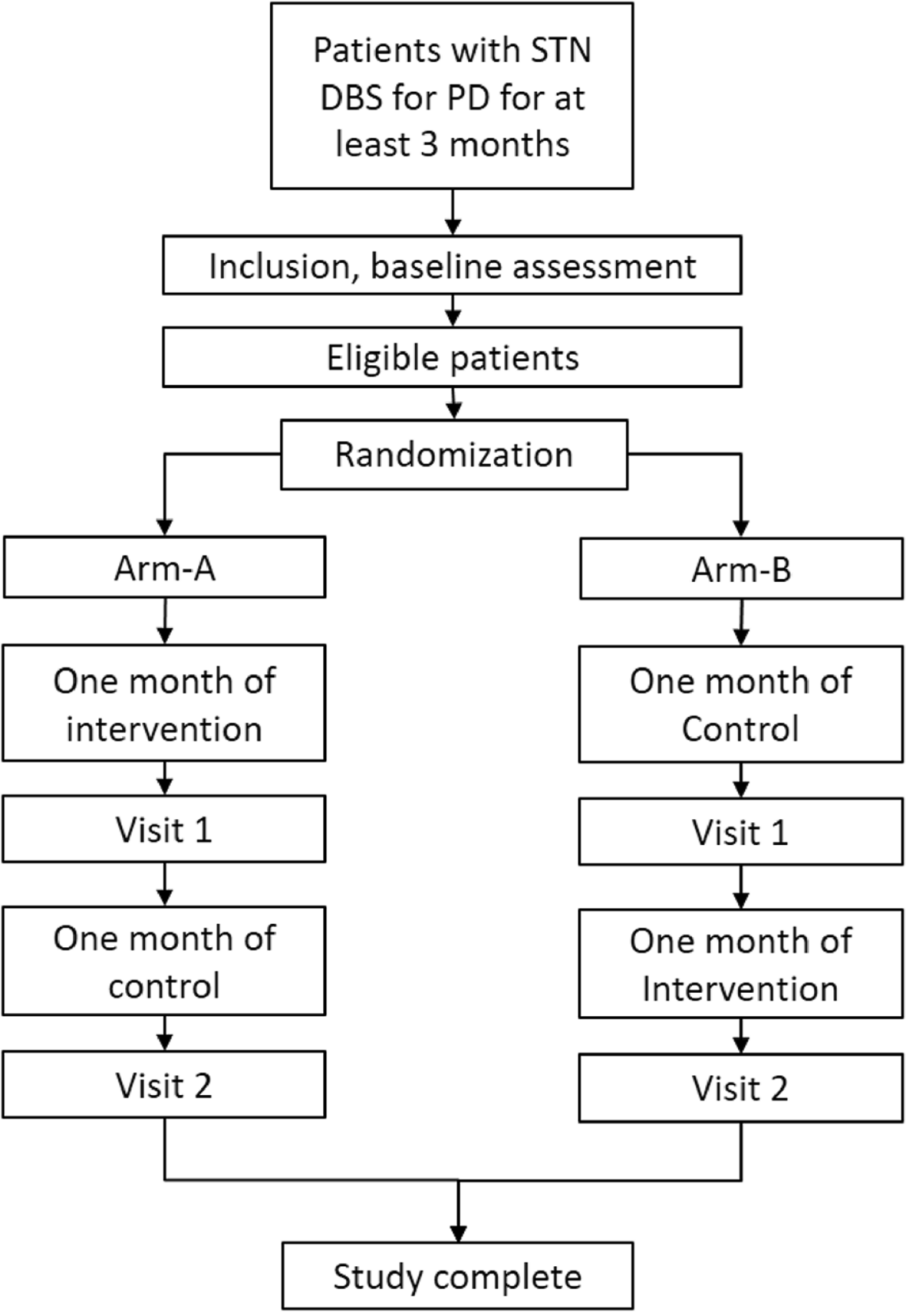
Inclusion and randomization

In this study, we will determine whether more dorsal stimulation of the STN decreases apathy compared to treatment as usual, including 26 PD patients suffering from apathy after ≥ 3 months of STN DBS. The Consolidated Standards of Reporting Trials (CONSORT) study schedule is shown in Fig. 1. The participating centers are Amsterdam University Medical Centers (Amsterdam UMC), St. Elisabeth TweeSteden ziekenhuis (ETZ), and HagaZiekenhuis (HZ). The first participant was included in February 2020. The study protocol was approved by the Medical Ethics Committee of the Amsterdam UMC, and the study is registered at the Netherlands Trial Register (number NL8279), currently included in the International Clinical Trial Registry Platform.

### Eligibility criteria {10}

The inclusion criteria are as follows: (a) idiopathic PD, (b) at least 3 months of STN DBS treatment, and (c) a score of 14 or more points on the Starkstein Apathy Scale (SAS). Exclusion criteria are as follows: (a) perioperative intracerebral complications related to STN DBS surgery (e.g., bleeding or infection) inflicting permanent changes, (b) cognitive decline, as measured by a Montreal Cognitive Assessment (MoCA) score of 25 or less, (c) participants who are not sufficient in the Dutch language, (d) participants who are already stimulated on the most dorsal contact point on both electrodes, and (e) no written informed consent.

### Who will take informed consent {26a}

Participants will be included and randomized after they sign the informed consent form obtained by a nurse practitioner or clinician. A nonprofessional caregiver, if one is available for the participant, will be asked to fill in informant-rated questionnaires at every timepoint.

### Additional consent provisions for collection and use of participant data and biological specimens {26b}

This trial does not involve collecting biological specimens for storage.

## Interventions

### Explanation for the choice of comparators {6b}

In an earlier case study, we presented three patients with severe apathy that were treated by switching stimulation from a ventral electrode contact point to a more dorsal contact point [18]. This increase in apathy after DBS was possibly related to collateral stimulation of limbic fibers [16, 17].

### Intervention description {11a}

Every DBS patient programmer will be installed with three programs; one is the escape option and will contain the original stimulation settings, and the other two contain either the investigational or the control settings. The investigational program stimulates one stimulation point more dorsally on the side with the most ventral active contact point relative to the AC-PC line based on the fused preoperative MRI and postoperative CT-scan images. The total duration of the visits of each included subject is 20–30 min per visit, for a total of three visits in 2 months (Table 1).

**Table 1:** Assessment schedule

	Pre-operative	Post-operative	Inclusion	Baseline	Visit 1 (+ 1 month)	Visit 2 (+ 2 months)	End of trial
SAS	X		X	X	X	X	
MRI, DTI	X						
CT scan		X					
In- and exclusion criteria			X				
MOCA			X				
Baseline characteristics				X			
MDS-UPDRS-III	X			X	X	X	
PDQ-39				X	X	X	
QUIP	X			X	X	X	
LEDD	X			X	X	X	
MADRS	X			X	X	X	
AES-I*				X	X	X	
SF-36*				X	X	X	
Suspected arm							X
Preferred settings							X

*SAS* Starkstein’s Apathy Scale, *MOCA* Montreal Cognitive Assessment, *MDS-UPDRS-III* Movement Disorder Society’s Unified Parkinson’s Disease Rating Scale, motor part III, *MADRS* Montgomery-Åsberg Depression Rating Scale (MADRS), *PDQ-39* 39-item Parkinson’s disease questionnaire, *QUIP* Parkinson's Disease Impulsive-Compulsive Disorders Questionnaire, *LEDD* levodopa-equivalent daily dosage, *AES-I* Apathy Evaluation Scale, a second apathy scale rated by the informal caregiver if the patient has one. *SF-36* Short-Form Health Survey. Suspected arm, patients will be asked to choose which arm they think they were randomized for. Preferred settings, patients will be asked to choose which of the settings they will continue with. *Although the timing for these questionnaires is the same as the visits for the patients, these questionnaires might be sent by regular mail to the informed caregiver

### Criteria for discontinuing or modifying allocated interventions {11b}

Participants are allowed to change the stimulation by plus or minus 0.5 V or milliampere.

### Strategies to improve adherence to interventions {11c}

To improve adherence to intervention protocol, the blinded assessor will check whether the programs were switched by the participant during the trial.

### Relevant concomitant care permitted or prohibited during the trial {11d}

No changes in LEDD are permitted during this study, and other concomitant care is not restricted during the course of this trial.

### Provisions for posttrial care {30}

Participants are able to continue with their preferred DBS stimulation settings after the trial and are referred back to their neurologist.

### Outcomes {12}

The primary outcome is the comparison of the Starkstein Apathy Score (SAS) score following 1 month of DBS on the original contact and the SAS score following 1 month of DBS on the more dorsal contact [19]. Secondary outcomes are symptom changes on the Movement Disorders Society-Unified Parkinson’s Disease Rating Scale motor part III (MDS-UPDRS-III) [20]. Montgomery-Åsberg Depression Rating Scale (MADRS) [21]. 39-item Parkinson’s disease questionnaire (PDQ-39) [22]. Parkinson’s Disease Impulsive-Compulsive Disorders Questionnaire (QUIP) [23]. changes in levodopa-equivalent daily dosage (LEDD) [24]. apathy rated by the caregiver (AES-I) [25]. and burden and quality of life of the caregiver (SF-36) [26]. Additionally, participants will be asked which program they thought contained the investigational settings (i.e., “first,” “second,” or “unsure”) and which program they wish to continue to use (i.e., first or second). The preoperative MRI, postoperative CT-scan, and preoperative SAS scores will be retrospectively collected to provide supplementary information about the relationship between apathy and stimulation location.

### Participant timeline {13}

The peri- and postoperative care of DBS surgery has been reported previously [27–29]. Although there are some differences among the centers, for example, the amount of microelectrode recordings, we expect no relevance for our study. Participants receive postoperative care by their neurologist and nurse practitioners and are screened for apathy or other motor and non-motor outcomes at least 3 months post-operatively or earlier when necessary. If a patient experiences symptoms of apathy, the neurologist, nurse practitioner, or consulting psychiatrist will check whether the patient meets the inclusion and exclusion criteria. Baseline assessments will be performed by their regular nurse practitioner, and subsequently, participants will be randomized by one of the coordinating investigator or co-investigators. A DBS clinician will determine the most ventral stimulating electrode relative to the AC-PC line, and the nurse practitioner will install the investigational settings in the patient programmer. The participant will either receive 1 month of investigational settings followed by 1 month of controlled settings or the opposite order, and the primary and secondary outcomes will be assessed by a blinded assessor.

### Sample size {14}

We estimated that a mean change of 8 points on the SAS is clinically relevant based on three cases in our center and two other studies that focused on the treatment of apathy in PD [14, 18, 30]. For our power calculation, we calculated that the two-sided *t*-test will achieve 80% power to ascertain that the mean difference is not 0 SAS points, if the total sample size of a two-by-two cross-over design is 20. If the actual mean difference between the DBS settings is 8 SAS points, the standard deviation of the paired differences is 12, and the significance level is 0.05, and 21 patients are needed to reach statistical significance. We aim to include a total of 26 patients because we anticipate a dropout up to 20%, because apathy may be difficult to combine with participation in a time-demanding clinical trial.

### Recruitment {15}

Based on the number of patients operated every year (40–80), we estimate that the inclusion of 26 patients should be feasible [8].

## Assignment of interventions: allocation

### Sequence generation {16a}

Participants will be randomized (1:1) to arm A and B using website-based randomization.

### Concealment mechanism {16b}

The blinded assessor is a second nurse practitioner, who will perform the 1- and 2-month follow-up assessment. The blinded assessor will not be able to access the stimulation settings of the programmer but will be able to switch from the first to the second program after 1 month.

### Implementation {16c}

The randomization will be performed by one of the coordinating investigator or co-investigators in Castor, Electronic Data Capture, to conceal intervention allocation [31]. Participants will only be randomized after the baseline measurements have been registered.

## Assignment of interventions: blinding

### Who will be blinded {17a}

Both the participant and the assessor will be blinded in this trial.

### Procedure for unblinding if needed {17b}

Individual treatment codes will be available to the involved neurologist at the study center for breaking the blind for medical emergencies necessitating treatment randomization.

## Data collection and management

### Plans for assessment and collection of outcomes {18a}

The assessors of each participating center are trained by the coordinating center (Amsterdam UMC) for the assessment of the study instruments. The study instruments involve the following questionnaires: SAS, with acceptable validity [32]. MDS-UPDRS-III, with strong concurrent validity [20]. MADRS with good validity and reliability [33]. PDQ-39 with good validity and acceptable reliability [34]. QUIP with good validity [35]. AES-I with good reliability [32]. and SF-36 with good reliability and validity [36].

### Plans to promote participant retention and complete follow-up {18b}

Participants who wish to discontinue the study are allowed to quit at any time, and the reason for withdrawal will be recorded if possible.

### Data management {19}

The data collection forms can be requested in the form of digital forms or physical case report forms. Missing SAS values will be analyzed as missing or as imputed using multiple imputation using baseline covariates as independent variables, and other missing data will be deleted listwise.

### Confidentiality {27}

All data concerning the subjects will be stored anonymously by a code that is unique for each subject. A subject identification code list will be kept by the principal investigator. The handling of personal data will comply with the Dutch Personal Data Protection Act. The local research institution will keep the source data for 15 years.

### Plans for collection, laboratory evaluation, and storage of biological specimens for genetic or molecular analysis in this trial/future use {33}

There will be no biological specimens collected.

## Statistical methods

### Statistical methods for primary and secondary outcomes {20a}

The difference in SAS scores between dorsal DBS and standard care will be examined using a mixed-effects model with a random intercept per patient. The SAS scores will be dichotomized using the proposed cutoff of 14. The *χ*^2^ test will be used to determine whether there is a difference in apathetic individuals between the investigational phase and the control phase. All statistical programming and analysis will be performed using IBM SPSS statistics version 24 (IBM Corp., Armonk, NY, USA).

### Interim analyses {21b}

No formal interim analysis on efficacy is planned.

### Methods for additional analyses {20b}

The SAS follow-up scores will be further investigated using McNemar tests, paired *t*-tests, and Wilcoxon signed-rank tests taking into account patients’ MDS-UPDRS-III, SAS, MADRS, QUIP, LEDD, PDQ-39, AES-I, and SF-36.

### Methods in analysis to handle protocol nonadherence and any statistical methods to handle missing data {20c}

The main statistical analyses of the primary endpoint will be based on the intention-to-treat principle. In addition, we will perform a per-protocol analysis on data from patients with complete SAS data from every time point.

### Plans to give access to the full protocol, participant-level data, and statistical code {31c}

De-identified participant data and statistical code are available on reasonable request, as is the full protocol.

## Oversight and monitoring

### Composition of the coordinating center and trial steering committee {5d}

The Amsterdam UMC center will coordinate the trial with day-to-day support for all centers with monthly meetings.

### Composition of the data monitoring committee and its role and reporting structure {21a}

There is no data and safety monitoring board because of the low expectance of safety concerns or risks for participants.

### Adverse event reporting and harms {22}

All adverse events reported spontaneously by the subject or observed by the investigator will be recorded and reported to the Medical Ethical Committee as required indicating expectedness, seriousness, severity, and causality.

### Frequency and plans for auditing trial conduct {23}

The independent Amsterdam UMC monitoring program will monitor this study for each participating center.

### Plans for communicating important protocol amendments {25}

The Medical Ethical Committee will be informed for any new protocol amendments and will be asked for approval.

### Dissemination plans {31a}

After completion of the study and data analysis, results will be made publically without restriction, independent of the outcome.

## Discussion

Patients suffering from PD often experience neuropsychiatric symptoms, which might even be the first detectable symptom of this neurodegenerative process [37]. DBS has great potential to relieve motor symptoms and may also have a beneficial effect on many non-motor symptoms [3, 27, 38]. One of the main challenges in the management of advanced PD and STN DBS is increased apathy after STN DBS with a high impact on quality of life [7]. The pathophysiology of apathy in PD, and particularly in the case of STN DBS, is still not fully understood; however, the relation between apathy and medication reduction and a direct stimulation effect are recognized as potential factors [9]. Because our proposed intervention is only a minor adjustment in the stimulation settings, this adjustment has the potential to improve the quality of life of many PD patients relying on STN DBS without many side effects. The application of STN DBS may subsequently be expanded as a patient’s mood might benefit from the treatment of invalidating PD motor and hypodopaminergic non-motor symptoms, as well as the possible reduction of hyperdopaminergic side effects that are allowed by lowered LEDD following STN DBS. The reduction of apathy might further increase physical health of people with PD who might have been limited in their activities by apathy and improve their social life. The study design has some limitations: first, the contact point that will be selected for the intervention will not be based on MRI imaging. This practice has been chosen because of the concept generalization; in this way, our intervention could be applied regardless of access to visualization of the STN subregions which are difficult to distinguish. The imaging of these subregions would preferably be done requiring imaging instruments on the level of 7-Tesla MRI scans, which is almost exclusively used for research and not clinical practice. Another argument for our decision was the desire to not influence the selection of intervention based on our hypothesis but to apply the intervention that has worked for previous patients [18]. However, this contact point was not necessarily closest to the motor region of the STN. Benefits of choosing the contact closest to the motor region of the STN on MRI imaging might have been the following: a more optimal motor response with possibly less required adjustments in LEDD or DBS current and less risk of stimulating adjacent regions with possible additional side effects. Second, we chose an apathy score of 14 or more on the SAS as inclusion criteria instead of an increased SAS score between pre- and postoperative assessments, which would more likely indicate solely STN DBS-induced apathy. Our argument for this decision was also generalizability because STN DBS-treated patients with apathy will have rarely available preoperative apathy scores for comparison. Furthermore, other studies found that STN DBS improved apathy or PD-related dysphoria without an earlier increase of apathy following STN DBS [39, 40]. Third, this study focused on apathy, although apathy is associated with other disorders. For example, apathy and depression share many features and might have some common pathophysiology when related to PD. We chose to include the most common disorders as secondary outcomes and not exclude participants with a current depressive or psychotic episode [3, 9, 41, 42]. Fourth, enrollment of participants was possible after only 3 months of STN DBS which might arguably be too soon because many centers optimize DBS stimulation and dopaminergic medication in the first 6 months. We chose the timepoint of at least 3 months because of four reasons: optimization of DBS and medication within 3 months are prioritized in our center, an earlier study showed that apathy did not further increase after 3 months [43]. cognitive decline might develop in the timespan of DBS surgery and optimization, and waiting on drugs for the treatment of depressive episodes to have effect was deemed inconsequential because this study aimed to treat apathy regardless of depressive symptoms. Fifth, 1 month of intervention is relatively short, we opted for this period because in our case study, and the direct stimulation effect was resolved within days to weeks [18]. This fast effect was also present in another case study [7]. To our knowledge, these are the only intervention reports on an imaging-supported STN DBS stimulation effect on apathy. However, relative to most non-motor symptoms and psychiatric disorders, an intervention of 1 month is short, and this could restrict the effectiveness of the trial. We hypothesize that switching stimulation to the motor region of the STN could be an optimization for DBS in PD, with an effect on multiple psychiatric symptoms and quality of life.

## Trial status

Recruitment started in February 2020 and is ongoing. Currently, 16 out of 26 participants have been included and finished follow-up (15th of January 2024).

Amendments are drafted after notifying the sponsor of changes to the protocol. The PI will notify the centers after approval by the Medical Ethical Committee and send a revised protocol to add to the Investigator Site File and the Clinical Trial Registry. Any deviations from the protocol will be fully documented using a protocol deviation form. The funder is notified yearly on the status of the trial including submitted amendments. Current approved amendments are as follows:

1. Amendment 1.1 was submitted on 05 September 2019 and specified the assessment of the most ventrally stimulating electrode based on fused preoperative MRI and postoperative CT-scan imaging.
2. Amendment 1.2 was submitted on 08 November 2019 by allowing for the inclusion of participants after 3 months after DBS surgery instead of 6 months and by allowing voltage or amperage adjustment during the study.
3. Amendment 1.3 was submitted on 13 April 2020 and stated an endpoint on the SAE reporting until 31 days after the last visit of the last participant on the advice of the monitor.

### Supplementary Information

**Additional file 1.** Study protocol version 1.3.

**Additional file 2:** **Figure 1.** Inclusion and randomization.

**Additional file 3:** **Table 1.** Assessment Schedule.

**Additional file 4.** World Health Trial Registration Data Set.

**Additional file 5.** SPIRIT schedule of enrollment, interventions and assessments.

**Additional file 6.** SPIRIT 2013 Checklist: Recommended items to address in a clinical trial protocol and related documents.

**Additional file 7.** SPIRIT Checklist for *Trials.*

**Additional file 8.** Model consent form (Dutch).

## Data Availability

De-identified participant data and statistical code are available on reasonable request. Springer Nature remains neutral with regard to jurisdictional claims in published maps and institutional affiliations.

## Abbreviations

Amsterdam UMC: Amsterdam University Medical Centers
STN: Subthalamic nucleus
DBS: Deep brain stimulation
SAS: Starkstein Apathy Scale
MDS-UPDRS: Modified Unified Parkinson’s Disease Rating Scale
METC: Medisch Ethische Toetsingscommissie (Medical Ethical Committee)
PD: Parkinson’s disease
