# Supplementary material for: A multicenter double-blind randomized crossover study comparing the impact of dorsal subthalamic nucleus deep brain stimulation versus standard care on apathy in Parkinson’s disease: a study protocol
Source: Trials. 2024 Feb 3;25:104. doi: 10.1186/s13063-024-07938-9 (PMC10837902; doi:10.1186/s13063-024-07938-9)
Supplement: Supplementary file 1 — Additional file 1. Study protocol version 1.3. [file 13063_2024_7938_MOESM1_ESM.pdf]

A multicenter double blind randomized crossover study comparing the impact of dorsal versus ventral subthalamic nucleus deep brain stimulation on apathy in Parkinson’s disease

(April 2020)

A multicenter double blind randomized crossover study comparing the impact of dorsal versus ventral subthalamic nucleus deep brain stimulation on apathy in Parkinson's disease

**Protocol ID**	2019-R3
**Short title**	Apathy and DBS in Parkinson's disease
**Version**	1.3
**Date**	13-04-2020
**Coordinating investigator**	Drs. T.J.C. Zoon<br>Amsterdam UMC, location AMC, Department of Psychiatry<br>Meibergdreef 5 1100 DD Amsterdam<br>t.j.zoon@amc.uva.nl
**Principal investigator**<br><br><br><br>**Co-principal investigator**	Prof. Dr. R.M.A. de Bie<br>Amsterdam UMC, location AMC, Department of Neurology<br>r.m.debie@amc.uva.nl<br>Prof. Dr. D.A.J.P. Denys<br>Amsterdam UMC, location AMC, Department of Psychiatry<br>d.denys@amc.uva.nl
**Co-investigators**	Prof. Dr. P.R. Schuurman<br>Amsterdam UMC, Department of Neurosurgery<br>e-mail p.r.schuurman@amc.uva.nl<br>Dr. P. van den Munckhof<br>Amsterdam UMC, Department of Neurosurgery<br>e-mail p.vandenmunckhof@amc.uva.nl<br>Dr. A.F. van Rootselaar<br>Amsterdam UMC, Department of Neurology<br>a.f.vanrootselaar@amc.uva.nl<br>Dr. G. van Rooijen<br>Amsterdam UMC, Department of Psychiatry<br>e-mail g.vanrooijen@amc.uva.nl<br>Dr. M.F. Contarino<br>HAGA Hospital The Hague

	m.contarino@hagaziekenhuis.nl<br>Dr. J.Th. van Asseldonk<br>St Elisabeth Hospital Tilburg<br>t.v.asseldonk@elisabeth.nl
**Sponsor**	Amsterdam UMC, location AMC, Department of Psychiatry<br>Meibergdreef 5, 1105 AZ Amsterdam
**Subsidising party**	Parkinson Vereniging Nederland
**Laboratory sites**	Amsterdam UMC, location AMC, Department of Neurology<br>Meibergdreef 9, 1105 AZ Amsterdam<br>HagaZiekenhuis<br>Els Borst-Eilersplein 275, 2545 AA den Haag<br>St. Elisabeth Ziekenhuis Tilburg<br>Hilvarenbeekse Weg 60, 5022 GC Tilburg

PROTOCOL SIGNATURE SHEET

Name	Signature	Date
Vice-Head of Department:<br>Prof. Dr. Y.B.W.E.M. Roos		
Principal Investigator:<br>Prof. Dr. R.M.A. de Bie		

TABLE OF CONTENTS

1. INTRODUCTION AND RATIONALE 9
2. OBJECTIVES 11
3. STUDY DESIGN 12
4. STUDY POPULATION 16
5. METHODS 18
5.1 Study parameters/endpoints 18
5.2 Study procedures 19
6. SAFETY REPORTING 23
6.1 Temporary halt for reasons of subject safety 23
6.2 AE's SAE's and SUSAR's 23
7. STATISTICAL ANALYSIS 26
7.1 Primary study parameters 26
7.2 Secondary study parameters 26
8. ETHICAL CONSIDERATIONS 28
8.1 Regulation statement 28
8.2 Recruitment and consent 28
8.3 Benefits and risks assessment, group relatedness 28
8.4 Compensation for injury 29
8.5 Incentives 29
9. ADMINISTRATIVE ASPECTS, MONITORING AND PUBLICATION 30
9.1 Handling and storage of data and documents 30
9.2 Monitoring and Quality Assurance 30
9.3 Amendments 30
9.4 Annual progress report 30
9.5 Temporary halt and (prematurely) end of study report 30
9.6 Public disclosure and publication policy 31
10. STRUCTURED RISK ANALYSIS 32
10.1 Potential issues of concern 32
10.2 Synthesis 33
11. REFERENCES 34

LIST OF ABBREVIATIONS AND RELEVANT DEFINITIONS

**AE**	**Adverse Event**
**AES-I**	**Apathy Evaluation Scale - informant rated**
**AR**	**Adverse Reaction**
**DBS**	**Deep Brain Stimulation**
**DTI**	**Diffusion Tensor Imaging**
**EPI**	**Echo-planar imaging**
**LEDD**	**Levodopa Equivalent Daily Dosage**
**MD**	**Medical Doctor**
**MOCA**	**Montreal Cognitive Assessment**
**NPO**	**Assessment of cognitive decline (in Dutch: Neuropsychologisch onderzoek)**
**PD**	**Parkinson's Disease**
**PDQ-39**	**39-item Parkinson's disease Questionnaire**
**PIL**	**Patient Information Leaflet**
**(S)AE**	**(Serious) Adverse Event**
**SF-36**	**Short-Form Health Survey**
**SAS**	**Starkstein Apathy Scale**
**STN**	**Subthalamic Nucleus**
**MDS-UPDRS-III**	**Movement Disorder Society's Unified Parkinson's disease Rating Scale (motor part III only)**
**WMO**	**Medical Research Involving Human Subjects Act (in Dutch: Wet Medisch-wetenschappelijk Onderzoek met Mensen**

SUMMARY

Rationale:
Deep brain stimulation (DBS) of the subthalamics nucleus (STN) is an effective treatment for advanced Parkinson's disease (PD). The prevalence of apathy — *i.e.,* loss of motivation and energy — after STN DBS ranges between 21% and 71%. Apathy after STN DBS may be caused by stimulation of the ventral STN, which connects with limbic circuits that have a role in motivation.

Objective:
To test the hypothesis that apathy after STN DBS may be reversed by switching the activated contact on the DBS-electrode from a ventral to a more dorsal contact.

Study design:
A multicenter double blind randomized crossover study in PD patients with STN DBS, comparing severity of apathy between the experimental dorsal stimulation setting and the original stimulation settings.

Study population:
26 PD patients with a score of 14 points or more on the Starkstein Apathy Scale (SAS) with at least three months of STN DBS.

Intervention:
26 PD patients will be randomly assigned to one of two arms. Arm-A will undergo 1 month of dorsal stimulation (intervention) followed by 1 month of regular stimulation (control). Arm-B will undergo 1 month of regular stimulation followed by 1 month of dorsal stimulation.

Main study parameters/endpoints:
The primary outcome is the comparison of the SAS score following one month of DBS on the original contact and the SAS score following one month of DBS on the more dorsal contact. Secondary outcomes are symptom changes on the Movement Disorders Society-Unified Parkinson's Disease Rating Scale motor part III (MDS-UPDRS-III), Montgomery- Åsberg Depression Rating Scale (MADRS), 39-item Parkinson's disease Questionnaire (PDQ-39) Parkinson's Disease Impulsive-Compulsive Disorders Questionnaire (QUIP), changes in levodopa-equivalent daily dosage (LEDD), apathy rated by the caregiver (AES-I), and burden and quality of life of the caregiver (SF-36). Imaging data available from standard clinical care (pre-operative structural and diffusion-weighted MRI scans and post-operative CT-scan) will

be used to determine the locations and white matter connections of the experimental and original stimulation contact, and to correlate this contact-specific neuroanatomical information with apathy scores and effectiveness of the intervention.

Nature and extent of the burden and risks associated with participation, benefit and group relatedness:

The burden of adjusting the DBS settings may include cognitive, affective, behavioural, and motor adverse effects. Severe adverse effects are unlikely. An escape option for both phases will be installed and support to switch to this option will be available at all times. The voltage or current of the DBS can be increased or decreased to adjust for the new settings when patients experience motor symptoms.

The experimental electrode contact is closely situated to the original contact and patients have already been stimulated at all contacts, including the experimental, during the optimization process of the DBS. The expected burden of the questionnaires is minor and the total time of the visits will be approximately 1 hour.

Patients included in this study will be offered the opportunity to continue DBS with the experimental settings if beneficial. The final aim of this study is to improve the quality of life of a large group of patients relying on DBS for PD.

1. INTRODUCTION AND RATIONALE

Deep Brain Stimulation (DBS) of the subthalamic nucleus (STN) is part of the standard care for advanced Parkinson's disease (PD).(1) Multiple studies have shown that STN DBS is able to reduce motor symptoms by on average 50 percent. (2,3) After implantation of the DBS-system, the stimulation parameters are optimized for the best improvement of motor symptoms, and for the assessment of symptom severity the Movement Disorders Society Unified Parkinson's Disease Rating Scale, motor part III (MDS-UPDRS-III) is used. Non-motor symptoms are less often assessed in standardized way and may be less often recognized because the symptoms take time to develop and are harder to recognize by both the patient and the clinician. STN DBS has some negative effect on a few intellectual abilities, such as fluency.(4,5) However, the increase of apathy is less often studied and has a potentially great negative effect on the patient's quality of life. (6) Apathy is described as a loss of motivation with decreased initiative, interest and energy and an emotional indifference with flat affect. (7)

We found 16 studies that measured the severity of apathy after STN DBS, of which 13 reported an increase of apathy despite motor improvement. (6,8,9) Based on nine studies using the Starkstein's proposed cut-off by for apathy, the point prevalence of apathy was approximately 46%, ranging between 21% and 71%. These studies suggest that apathy may be a common adverse effect of STN DBS and an important trade-off for patients suffering from advanced PD and relying on this treatment. Apathy in PD is associated with the lowest quality of life compared to all other PD symptoms. (10) A recent study showed that patients suffering from apathy after STN DBS did not experience improved quality of life despite improvement of motor symptoms. (9)

One explanation for the occurrence of apathy after STN-DBS is the necessary reduction of dopaminergic medication after STN-DBS. However, longitudinal studies do not consistently show correlation between apathy and decreased levodopa-equivalent daily dosage (LEDD) (9,11). Two recent neuroimaging studies suggest an association between apathy after STN DBS and stimulation of the ventral part of the STN, associated with non-motor limbic circuits involved in emotion regulation and motivation. (8,12) The STN is a relatively small brain structure with three regions that separately connect to motor, limbic, and cognitive circuits. As these neuronal tracks are closely situated to each other and their projections partly overlap, stimulating the motor region with STN DBS may also influence limbic and cognitive circuits. The effect of STN DBS on motor symptoms is directly observable during parameter optimization, while cognitive, affective and behavioral effects may become apparent only

after several months and may not be detected during the optimization process. Furthermore, advanced Parkinson's disease with severe motor and non-motor is also known to be a burden on the informal caregivers. The quality of life of these important individuals will be assessed during the intervention trial, as well as an informant based apathy scale to provide hetero-anamnestic information. In the Amsterdam University Medical Centers in Amsterdam, we have described at least three cases of post-operative apathy after DBS, which improved immediately after switching the parameters to activate a more dorsal contact on the STN-electrode. (15).

In the current study, we will test whether STN DBS related apathy could be reversed by switching stimulation from a ventral to a more dorsal contact on the electrode. We propose a multicenter double blind randomized crossover study including 26 PD patients with apathy after STN DBS surgery. We expect that the results of this study can help to improve the quality of life of many PD patients by providing a possible treatment for apathy associated with STN DBS. In addition, the results could provide insight in the pathophysiological substrate for apathy and can be used for further investigation in this field of research.

In summary, this study will test whether switching stimulation to a more dorsal region of the STN will result in decreased severity of apathy. Patients scoring positive on the SAS with 3 months of DBS will be included and randomized into an intervention and a control phase in a double-blinded fashion.

2. OBJECTIVES

Primary Objective:
To test the hypothesis that apathy after STN DBS may be reversed by switching the activated contact on the DBS-electrode from a ventral to a more dorsal contact.

Secondary Objectives:
1. To determine if apathy can be resolved with dorsal STN DBS without losing the optimal motor effects.
2. To measure the possible correlation between severity of apathy at baseline and baseline levodopa, dopamine-agonists and the combined daily dosage (LEDD) use.
3. To determine the effects of more dorsal STN DBS on depression, mania, impulsive and compulsive behaviours, observation of apathy by the caregiver and the quality of life of the caregiver.
4. To determine the neuroanatomical location and white matter connections of the experimental and original stimulation site, and to correlate this information with apathy scores.

3. STUDY DESIGN

3.1 Design

This study is a multicenter double blind randomized crossover study including 26 PD patients with apathy after at least 3 months of STN DBS. From April 2019 until September 2021, patients with apathy will be recruited for participation during a standard follow-up appointment by their neurologist, psychiatrist or specialized nurse practitioner in the Amsterdam UMC, location AMC, HagaZiekenhuis, and the St Elisabethziekenhuis. Apathy will be tested using the SAS, and patients scoring 14 or more will be asked to participate in this study if they meet the inclusion criteria. Eligible patients will be randomized (1:1) to one of two arms after signing informed consent and providing the contact information of an informal caregiver that provides the most care for the patient. This person cannot be a professional caregiver. It will be registered if the patient has no informal caregiver. The patient will be provided with the Patient Information Leaflet (PIL) providing information on the study. Because of the effect of the adjustments of the stimulation on the previously optimized motor symptoms, patients are more prone to identifying the intervention phase. To address this problem, the PIL will provide information about the possible side-effects of the intervention, without stating which side-effects are more likely to occur in each phase.
At this time, the DBS will be programmed for one of the two arms. Arm-A will undergo 1 month of dorsal stimulation (intervention) followed by 1 month of regular stimulation (control). Arm-B will undergo 1 month of regular stimulation followed by 1 month of dorsal stimulation. Each patient has two DBS-electrodes. The intervention will be done for one electrode only. The electrode of which the active contact is most ventrally located on fused images of the pre-surgery MRI and the postoperative CT-scan, using the anterior commissure – posterior commissure line for reference, will be chosen for the intervention. The intervention will consist of a program where the active contact will be switched to the closest dorsal location. The control phase will consist of a program with the original DBS settings.
The adjustment of ±0.5 milli ampere or ±0.5 volt is permitted to adjust for the new settings when patients experience side-effects of the treatment. The original setting will be programmed as an escape option for both phases. In case patients experience adverse effects, they will be seen on the outpatient clinic to optimize the power of the DBS or they will be guided by the investigator to switch back to their original settings without breaking the blind. The DBS in all patients will be set up with three programs, of which program 1 will always be the escape option with the regular settings. Programs 2 and 3 will contain either the control or the intervention settings and these will be programmed at the start of the study. After 1 month, with a range of -2 or +2 weeks, all patients will be contacted to fill in the questionnaires after which patients will enter the cross-over phase: the patients in the

intervention phase of the last month will receive the control settings and the control phase will receive the intervention settings. The patients and the investigator will be blind for the conditions.

The clinical scores (SAS, MDS-UPDRS-III, MADRS, PDQ-39, QUIP, and LEDD) and the informant scales (AES-I and SF-36) obtained after each phase, will be used to analyze the difference in apathy and secondary clinical outcomes between the intervention and control phase. The informant rated scales will be send by regular mail to the informal caregiver. The MDS-UPDRS-III will be performed during the OFF period of medication while the DBS is ON, as the best presentation of the function of the DBS. In addition, imaging data available from standard clinical care (pre-operative MRI and DWI scans and post-operative CT-scan) will be used to determine the exact locations and white matter connections of the experimental and original stimulation site, and to correlate contact-specific location and networks with apathy scores.

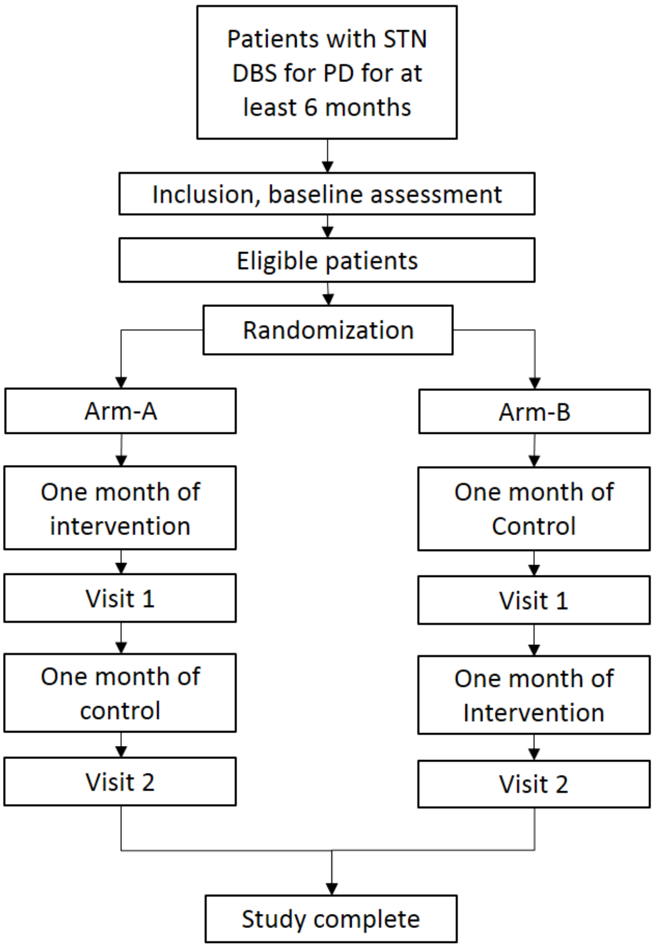

Figure 1.
Study design

3.2 Duration

The total duration of the visits of each included subject is 60 minutes divided over 3 visits in two months. (Table 1.)

	Pre-operative	Post-operative	Inclusion	Baseline	Visit 1 (+1 month)	Visit 2 (+2 months)	End of trial
SAS	X		X	X	X	X	
MRI, DTI	X						
CT-scan		X					
In- and ex-clusioncriteria			X				
MOCA			X				
Baseline characteristics				X			
MDS-UPDRS-III	X			X	X	X	
PDQ-39				X	X	X	
QUIP	X			X	X	X	
LEDD	X			X	X	X	
MADRS	X			X	X	X	
AES-I*				X	X	X	
SF-36*				X	X	X	
Suspected Arm							X
Preferred settings							X

Table 1. Assessment Schedule

SAS: Starkstein's Apathy Scale. MOCA: Montreal Cognitive Assessment. MDS-UPDRS-III: Movement Disorder Society's Unified Parkinson's Disease Rating Scale, motor part III. MADRS: Montgomery- Åsberg Depression Rating Scale (MADRS). PDQ-39: 39-item Parkinson's disease Questionnaire. QUIP: Parkinson's Disease Impulsive-Compulsive Disorders Questionnaire. LEDD: levodopa-equivalent daily dosage. AES-I: Apathy Evaluation Scale, a second apathy scale rated by the informal caregiver if the patient has one. SF-36: Short-Form Health Survey. Suspected Arm: Patients will be asked to choose which arm they

think they were randomized for. Preferred Settings: Patients will be asked to choose which of the settings they will continue with. *Although the timing for these questionnaires is the same as the visits for the patients, these questionnaires will be send by regular mail to the informed caregiver.

3.3 Setting

Participants will be recruited at the DBS neurology departments of the Amsterdam UMC, Hagaziekenhuis and St Elisabethziekenhuis. All measurements will be performed at the hospital where the patients received the care for the STN DBS and all analyses will be performed at the Amsterdam UMC. The questionnaires for the caregiver will be send from the Amsterdam UMC and the results will be gathered in the Amsterdam UMC.

4. STUDY POPULATION

4.1 Population

All patients with apathy after STN DBS in three centres will be approached for participation in this study by their treating nurse practitioner or physician. Having participated in another earlier clinical trials is not an exclusion criterion for participation in the current study.

4.2 Inclusion criteria

In order to be eligible to participate in the study, a subject must meet all of the following criteria:

1. Patients suffering from Parkinson's disease.
2. At least three months of STN DBS surgery.
3. Apathy — *i.e.*, a 14 or more points on the SAS in PD patients.

4.3 Exclusion criteria

1. Peri-operative intracerebral complications related to SNT-DBS placement (*e.g.*, bleeding or infection) inflicting permanent changes.
2. Dementia (MOCA score of 25 or less)
3. Patients who are not sufficient in the Dutch language
4. Patients who are already stimulated on the most dorsal contact point on both electrodes
4. Legally incompetent adults
5. No signed informed consent

4.4 sample size calculation

The primary outcome of this study is the difference in SAS score between the two stimulation settings. To the best of our knowledge, there are no published studies on the treatment of apathy, as measured using the SAS score, by changing the DBS simulation settings in PD patients treated with DBS. However, in our own case-study we describe three cases of apathy responding to the proposed intervention. (15) The baseline SAS score of these patients ranged between 21 and 30 points. The mean change in SAS score before and after the change in DBS simulation setting was 19 points with a standard deviation of the paired differences of 4.0 based on the measurements performed during the admission.

However, we will include patients with SAS scores from 14 points or more on the SAS in this study. As these patients may demonstrate a more variable response to the intervention, we conservatively assume that we will observe a mean change of 8 SAS points with a standard deviation of the paired differences of 12 points. We regard an improvement of 8 SAS points

as clinically relevant, based on the three patients in our case study who felt relieved of apathy after a decrease in score in this order of magnitude, as well as two studies where patients with apathy in Parkinson's disease were treated. (13,14,15) A two-sided t-test will achieve 80% power to infer that the mean difference is not 0 SAS points if the total sample size of a two-by-two cross-over design is 20. The actual mean difference between the DBS settings is 8 SAS points, the standard deviation of the paired differences is 12, and the significance level is 0.05. 21 patients are needed to reach statistical significance. As we expect up to 20% dropout in this study, we will aim to include a total of 26 patients in this study.

5. METHODS

5.1 Study parameters/endpoints

Main study endpoint

The main study parameter is the change in Starkstein Apathy Scale (SAS) score between regular and more dorsal stimulation. (16) A higher score on this questionnaire signifies more severe apathy with a cut-off of 14 points, corresponding with apathy.

Secondary study parameters

1. Some patients have SAS scores prior to the operation, these scores will be used to determine whether apathy was present before DBS. (16)
2. Motor PD symptom changes using the motor part (part III) of the MDS-Unified Parkinson's Disease Rating Scale (MDS-UPDRS-III). (17) A higher score is indicative of more symptoms of Parkinson's disease. This scale will be assessed during the OFF medication, ON DBS period.
3. Depression symptom changes using the Montgomery- Åsberg Depression Rating Scale (MADRS) with a score ranging from 0 to 60. A higher score is indicative of depressive symptoms, with a cut-off of 0-7 for the normal range and a score of 20 or higher for a moderate severity depression. (18)
4. Quality of life related to the symptoms of Parkinson's Disease using the 39-item Parkinson's disease Questionnaire (PDQ-39) with a score ranging from 0 to 100, a higher score indicates worse quality of life. (19)
5. Impulsive-Compulsive symptom changes using the Impulsive-Compulsive Disorders Questionnaire (QUIP) (20). The cutoff point for each subscale is (1) gambling: affirmative answers to 2 or more questions, (2) sexual behaviour: 1 or more questions; (3) buying: 1 or more questions; and (4) eating: 2 or more questions.
6. The relation between the severity of apathy changes and the change in dopaminergic medication (LEDD). A higher score means more levodopa equivalent medication usage. (21) Dopamine agonists (apomorphine, bromocriptine, rotigotine, pramipexole, ropinirole) will be included in the calculation and will also be registered independently.
7. The anatomical relationship between structural white matter tracts and the experimental and control contact will be determined on available pre- and post-DBS brain imaging scans. Merging the pre-operative DWI and structural MRI scans with the post-operative CT scan, tractography analysis will be performed to investigate the spatial relationship between the activated contact and local white matter tracts (such as the cortico-spinal tract, dentato-rubro-thalamic tract, medial forebrain bundle, internal capsule and medial

lemniscus. (22,23) This way, we aim to elucidate whether an eventual change in apathy and clinical effectiveness in more dorsal stimulation is related to a difference in stimulated circuitry.

8. Cognitive functioning will be assessed using the Montreal Cognitive Assessment (MOCA), a lower score means less cognitive function with a cut-off of 26 out of 30 possible points. (24)
9. At the end of the study, patients will be asked in which order they think they received the settings, this information can be used to determine whether an increase/decrease in apathy or an increase/decrease in motor symptoms influenced the blinding
10. At the end of the study, patients will be asked which settings they prefer, this will be registered to determine whether the patient preferred one program over the other, and might be used as a robust measurement of the weight of motor symptoms versus non-motor symptoms
11. The informal caregiver will be asked to fill in the Apathy Evaluation Scale - Informant (AES-I) with a score from 18-72, with a higher score indicating more severe apathy, the cut-off score for apathy is 38 or higher. This is used to provide hetero-anamnestic information about the apathetic behavior of the patient. This information is thought to be of importance as it is yet unclear whether apathetic patients are able to register apathy, and also whether the environment of the patient is aware of the non-motor symptoms of the patient. (25)
12. The informal caregiver will be asked to fill in the Short-Form Health Survey (SF-36) tomeasure the Quality of Life of the caregiver. This questionnaire uses a score ranging from 0-100 with a higher score indicating less impaired quality of life. (26)

5.2 Study procedures

a. Investigational STN DBS settings

When the neurologist evaluates a patient for eligibility at least 3 months after the operation, he will check the inclusion and exclusion criteria. The neurologist will introduce the study to the patient, inform the patient, and ask the patient permission to be contacted by a research nurse. If the patient is eligible and agrees, the neurologist will inform the research nurse at the Amsterdam UMC, location AMC. The research nurse at the AMC will register the patient in the database, after which the local research nurse will be informed. The neurologist will provide the patient with written information about the study and with the Informed Consent Form. The patient will be contacted by phone to answer any questions about the study. Patients will be given as much time as needed to decide if they want to participate. If necessary, an appointment will be made to answer any questions. If the patient agrees to participate, the first appointment will be made (Inclusion). At the start of the Inclusion, the

patient signs the informed consent form before continuation of the assessments. At the end of the first visit, the research nurse will randomize the patient by the central website based computer program. Patients will be randomized (1:1) to one of two arms. Arm-A will undergo 1 month of dorsal stimulation (intervention) followed by 1 month of regular stimulation (control). Arm-B will undergo 1 month of regular stimulation followed by 1 month of dorsal stimulation. The intervention will consist of a program where the active contact on the most ventrally activated electrode will be switched to one contact more dorsally without adjusting the frequency of voltage (please see 3.1). During the control phase, patients will be stimulated with the original DBS settings.

The DBS in all patients will be set up with three programs, of which program 1 will always be the escape option with the regular settings. Programs 2 and 3 will randomly contain either the control or the intervention settings and these will be programmed at the start of the study. During the study, the medication for Parkinson's disease (LEDD) will not be altered unless a (S)AE is reported. After 1 month, with a range of -2 or +2 weeks, all patients will be contacted to fill in the questionnaires after which patients will enter the cross-over phase: the patients in the intervention phase of the last month will receive the control settings and the control phase will receive the intervention settings. The DBS monitor will not show the stimulating contact and the DBS settings will be simply switched from program 2 to program 3 and vice versa without viewing the settings. As such, the patients and the investigator will be blind for the conditions (Figure 1). The programmed settings will be registered by the coordinating investigator, primary investigators and co-investigators and it is possible to consult in case of an (S)AE or for breaking the blind. Patients who feel severely discomforted can be treated by adjusting the power of the DBS and have the escape option to change the study settings to the original settings with the patient controller at home. A member of the researching team will be available to instruct the patients or the neurologist on site by telephone on how to activate program 1. At the end of the trial, the patients will be given the option to choose which settings they preferred without breaking the blind, this will be done in a shared decision making fashion with the neurologist or the nurse practitioner on site.

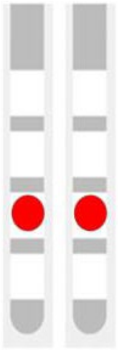

Figure 1.
Example of DBS settings with the two electrodes stimulated at the same contact point.
In that case, the post-surgery CT-scan will be used to determine which electrode is more ventrally positioned and the active contact point of that electrode will be set to 1 contact point more dorsally using the anterior commissure – posterior commissure line.

b. Randomisation, blinding and treatment allocation

Patients will be randomized to the treatment order 'experimental design DBS' followed by 'regular DBS' (*i.e.*, arm-A), or 'regular DBS' followed by 'experimental study design DBS' (*i.e.*, arm-B) in the ratio 1:1 using an random block design with blocks of size two and four. We will not stratify patients in the randomisation. We will randomise patients using Castor, Electronic Data Capture.
Every patient programmer will be installed with three programs by their nurse or neurologist; patients and the investigator will not be able to see the settings in each program and are blind in this way. Web-based code break will be provided for each patient indicating the investigational STN DBS settings blinding for both the investigator and the patient before the start of the intervention.

c. Methods for breaking the blind

Individual treatment codes, indicating the treatment randomization for each subject, will be available to the involved neurologist at the study centre. The treatment code will not be broken except in medical emergencies when the appropriate management of the subject necessitates knowledge of the treatment randomisation. Any breaking of the treatment code will be documented. Treatment codes will not be broken for the planned analyses of data until all decisions on the availability of the data from each individual subject have been made and documented.

d. Withdrawal of individual subjects

Subjects can leave the study at any time for any reason if they wish to do so without any consequences. The investigator can decide to withdraw a subject from the study for urgent medical reasons. Patients can refuse to let their data be included in the study. The reasons for discontinuation will be recorded if patients are willing to disclose this information.

e. Replacement of individual subjects after withdrawal

Subjects will not be replaced after withdrawal.

f. Follow-up of subjects withdrawn from treatment

All subjects will be analysed according to the intention to treat principle and follow up will be completed.

g. Premature termination of the study

No formal interim-analysis on efficacy is planned. Any mortality will be reported directly to the safety committee and evaluated for cause of death and possible trial related serious adverse events (SAEs). All SAEs will be reported online to the Central Committee on Research involving Human Subjects (CCMO) on www.ccmo.nl.

6. SAFETY REPORTING

6.1 Temporary halt for reasons of subject safety

In accordance to section 10, subsection 4, of the WMO, the sponsor will suspend the study if there is sufficient ground that continuation of the study will jeopardise subject health or safety. The sponsor will notify the accredited METC without undue delay of a temporary halt including the reason for such an action. The study will be suspended pending a further positive decision by the accredited METC. The investigator will take care that all subjects are kept informed.

6.2 AEs, SAEs and SUSARs

i. Adverse events (AEs)

Adverse events are defined as any undesirable experience occurring to a subject during the study, whether or not considered related to the experimental intervention. All adverse events reported spontaneously by the subject or observed by the investigator or his staff will be recorded.

ii. Serious adverse events (SAEs)

A serious adverse event is any untoward medical occurrence or effect that
- results in death;
- is life threatening (at the time of the event);
- requires hospitalisation or prolongation of existing inpatients' hospitalisation;
- results in persistent or significant disability or incapacity;
- is a congenital anomaly or birth defect; or
- any other important medical event that did not result in any of the outcomes listed above due to medical or surgical intervention but could have been based upon appropriate judgement by the investigator.

The following situations do not need to be reported as (S)AEs:
- Any admission unrelated to an AE, e.g. cosmetic surgery, social and/or convenience admissions to a hospital.
- Elective hospitalization (planned before the subject consented to study participation) for pre-existing conditions that have not been exacerbated by study treatment as judged by the clinical investigator and where admission did not take longer than anticipated.
- Admission for diagnosis or therapy of a condition that existed before inclusion to this

study and has not increased in severity or frequency as judged by the clinical investigator.
- Anticipated day-to-day fluctuations of pre-existing disease(s) or condition(s) present at the start of the study that do not worsen.
- Protocol-specified admission, e.g. for a procedure required by the study protocol.

The sponsor will report the SAEs through the web portal *ToetsingOnline* to the accredited METC that approved the protocol, within 7 days of first knowledge for SAEs that result in death or are life threatening followed by a period of maximum of 8 days to complete the initial preliminary report. SAEs will be reported from the moment that informed consent is given until 31 days after the last visit. All other SAEs will be reported within a period of maximum 15 days after the sponsor has first knowledge of the serious adverse events.

iii. Suspected unexpected serious adverse reactions (SUSARs)
Adverse reactions are all untoward and unintended responses to an investigational product related to any dose administered.
Unexpected adverse reactions are SUSARs if the following three conditions are met:
- the event must be serious
- there must be a certain degree of probability that the event is a harmful and an undesirable reaction to the medicinal product under investigation, regardless of the administered dose;
- the adverse reaction must be unexpected, that is to say, the nature and severity of the adverse reaction are not in agreement with the product information as recorded in:
- Summary of Product Characteristics (SPC) for an authorised medicinal product;
- Investigator's Brochure for an unauthorised medicinal product.

The sponsor will report expedited the following SUSARs through the web portal *ToetsingOnline* to the METC
- SUSARs that have arisen in the clinical trial that was assessed by the METC;
- SUSARs that have arisen in other clinical trials of the same sponsor and with the same medicinal product, and that could have consequences for the safety of the subjects involved in the clinical trial that was assessed by the METC.

The remaining SUSARs are recorded in an overview list (line-listing) that will be submitted once every half year to the METC. This line-listing provides an overview of all SUSARs from the study medicine, accompanied by a brief report highlighting the main points of concern. The expedited reporting of SUSARs through the web portal Eudravigilance or ToetsingOnline is sufficient as notification to the competent authority.

The sponsor will report expedited all SUSARs to the competent authorities in other Member States, according to the requirements of the Member States.
The expedited reporting will occur not later than 15 days after the sponsor has first knowledge of the adverse reactions. For fatal or life threatening cases the term will be maximal 7 days for a preliminary report with another 8 days for completion of the report.

c. Annual safety report

In addition to the expedited reporting of SUSARs, the sponsor will submit, once a year throughout the clinical trial, a safety report to the accredited METC, competent authority, and competent authorities of the concerned Member States.
This safety report consists of:

- a list of all suspected (unexpected or expected) serious adverse reactions, along with an aggregated summary table of all reported serious adverse reactions, ordered by organ system, per study;
- a report concerning the safety of the subjects, consisting of a complete safety analysis and an evaluation of the balance between the efficacy and the harmfulness of the medicine under investigation.
- All AEs will be followed until they have abated, or until a stable situation has been reached. Depending on the event, follow up may require additional tests or medical procedures as indicated, and/or referral to the general physician or a medical specialist.
- SAEs need to be reported till end of study within the Netherlands, as defined in the protocol.

7. STATISTICAL ANALYSIS

We will present baseline patient characteristics using descriptive statistics. We will use the mean and standard deviation to describe normally distributed continuous variables and the median and upper and lower limits of the interquartile range to describe non-normally distributed continuous variables. We will assess the normality of continuous variables by visually inspecting histograms. We will use counts and percentages to present categorical variables. No formal statistical testing will be performed to examine differences in baseline patient characteristics between the trial arms.

All statistical programming and analysis will be performed using IBM SPSS statistics version 24 (IBM Corp., Armonk. NY, USA). Two-sided p-values less than 0.05 will be considered statistically significant and statistical uncertainty will be expressed using two-sided 95% confidence intervals. Our reporting of the results will be consistent with relevant CONSORT guidelines.

7.1 Primary study parameters

The main statistical analyses of the primary endpoint will be based on the intention-to-treat principle. For the intention-to-treat analysis, missing SAS score values will be reported as missing for robustness. The difference in SAS scores between ventral and dorsal DBS will be examined using a mixed-effects model with a random intercept per patient. In addition, we will perform a per-protocol analysis on data from patients with complete SAS data from every time point.

7.2 Secondary study parameters

The SAS scores will be dichotomized using the proposed cut-off of 14. The X2 – test will be used to determine whether there is a difference in apathetic individuals between the investigational phase and the control phase.

7.3 Additional analyses

To gain further insight we will perform additional tests using the available data that is collected during this study. The SAS follow-up scores will be further investigated using McNemar tests, paired t-tests and Wilcoxon-signed rank tests taking into account patients' SAS scores and the MADRS to differentiate apathy from depression, the QUIP to determine impulsive behavior, and the LEDD to help differentiate apathy from a decrease in dopaminergic medication. The MDS-UPDRS-III will be used to measure motor function and to determine if a more suitable stimulation will have any effect on the motor symptoms of PD

and the PDQ-39 will be used to determine the quality of life. The AES-I will be used to provide a more uniform representation of apathy and the SF-36 will be used to measure the quality of life of the caregiver. Diffusion MRI scans will be analyzed using the appropriate toolboxes (ExploreDTI, FSL, MRtrix, Trackvis or Tracula (24). After preprocessing of the DWI data, which includes motion and eddy current artefact correction, we will perform tractography analysis based on a crossing-fiber model to identify ventral limbic and dorsal tracts within the volume of activated tissue (using the Lead-DBS toolbox(27)) of the experimental and control contacts, and the distance of these tracts to either of the contacts. Missing SAS values will be analyzed as missing or as imputed using multiple imputation using baseline covariates as independent variables.

8. ETHICAL CONSIDERATIONS

8.1 Regulation statement

This study will be conducted in accordance with the principles of the Declaration of Helsinki (5th Amendment, Edinburgh, 2000, with the notes of clarification of Washington, 2002 and Tokyo, 2004) and with the Medical Research Involving Human Subjects Act (WMO)

8.2 Recruitment and consent

Patients eligible for STN DBS will be asked to participate in the study during a post-operative visit as part of standard care by a psychiatrist, neurologist, psychiatrist in training, or a nurse practitioner specialized in Parkinson's disease. The principal investigator will ensure that the subject is given full and adequate oral and written information about the nature, purpose, possible risk and benefit of the study. Subjects must also be notified that they are free to discontinue at any time. The subject will be given the opportunity to ask questions and be allowed to consider the information provided. The subject's signed and dated informed consent must be obtained before conducting any procedure specifically for the study
When a subject would like to participate, the clinician will ensure that the subject is given full and adequate information about the nature, purpose, possible risk and benefit of the study. Subjects must also be notified that they are free to discontinue at any time and that they can be excluded from analysis. Objection by minors or incapacitated subjects will not be included. The patient will be asked to provide the contact information, *i.e. phone number and physical address,* of the informal caregiver. The informal caregiver will be asked consent by phone to provide additional information on apathy and the quality of life of the caregiver that is burdened by the care of the patient by filling in two questionnaires that are filled in and are mailed to them. If the patient has no informal caregiver or if the informal caregiver does not provide consent to participate, the patient will be included without the AES-I and the SF-36. When the informal caregiver does not answer on the first try, he or she will be contacted two more times by telephone. If the caregiver has not returned one or more questionnaires, he or she will be contacted by telephone one time.

8.3 Benefits and risks assessment, group relatedness

The major benefit for the participants in this study is a possible reduction in severity of apathy with a positive effect on the quality of life. The risks of adjusting the DBS settings in this study are likely to be minor. The most serious adverse event that may occur is a less than optimal motor improvement by the DBS with the experimental setting, which can be resolved by reprogramming the original settings. The burden for participating in the study and filling in questionnaires can be considered minimal. The SAS score will be provided to the participant

and can be used by the clinician to evaluate STN DBS settings or refer the patient for psychiatric evaluation.

8.4 Compensation for injury

The sponsor/investigator has a liability insurance, which is in accordance with article 7, subsection 9 of the WMO.
The sponsor (also) has insurance, which is in accordance with the legal requirements in the Netherlands (Article 7 WMO). This insurance provides cover for damage to research subjects through injury or death caused by the study. The insurance applies to the damage that becomes apparent during the study or within 4 years after the end of the study.
The sponsor/investigator has a liability insurance, which is in accordance with article 7 of the WMO.
The sponsor (also) has insurance, which is in accordance with the legal requirements in the Netherlands (Article 7 WMO). This insurance provides cover for damage to research subjects through injury or death caused by the study.
The insurance applies to the damage that becomes apparent during the study or within 4 years after the end of the study.

8.5 Incentives

There will likely be no incentives for the patients. The inclusion and the baseline will be performed during a standardized care visit. Patients will be compensated for the cost of the travel for the extra visits they will make.

9. ADMINISTRATIVE ASPECTS, MONITORING AND PUBLICATION

9.1 Handling and storage of data and documents

All data concerning the subjects will be stored anonymously by a code that is unique for each subject. A subject identification code list will be kept by the principal investigator. The handling of personal data will comply with the Dutch Personal Data Protection Act. The project leader will keep the source data for 15 years.

9.2 Monitoring and Quality Assurance

The Amsterdam UMC program will monitor this study as part of standard procedure for the use of medical equipment.

9.3 Amendments

A 'substantial amendment' is defined as an amendment to the terms of the METC application, or the protocol or any other supporting documentation, that is likely to affect to a significant degree:

- the safety or physical or mental integrity of the subjects of the trial;
- the scientific value of the trial;
- the conduct of management of the trial; or
- the quality or safety of any invention used in the trial.

All substantial amendments will be notified to the METC and to the competent authority.

Non-substantial amendments will not be notified to the accredited METC and the competent authority, but will be recorded and filed by the investigator/sponsor.

9.4 Annual progress report

The sponsor/investigator will submit a summary of the progress of the trial to the accredited METC once a year. Information will be provided on the date of inclusion of the first subject, numbers of subjects included and numbers of subjects that have completed the trial, serious adverse events/ serious adverse reactions, other problems, and amendments.

9.5 Temporary halt and (prematurely) end of study report

The sponsor will notify the accredited METC and the competent authority of the end of the study within a period of 90 days. The end of the study is defined as the last patient's last visit.

The sponsor will notify the METC immediately of a temporary halt of the study, including the reason of such an action.
In case the study is ended prematurely, the sponsor will notify the accredited METC and the competent authority within 15 days, including the reasons for the premature termination.
Within one year after the end of the study, the investigator/sponsor will submit a final study report with the results of the study, including any publications/abstracts of the study, to the accredited METC and the Competent Authority.

9.6 Public disclosure and publication policy

The trial will be registered online before start of inclusion. After completion of the study and analysis of the data results will be made publically without restriction, independent of the outcome. They will be submitted for publication to an international peer-reviewed journal.

10. STRUCTURED RISK ANALYSIS

10.1 Potential issues of concern

a. Previous exposure of human beings with the test product(s) and/or products with a similar biological mechanism

The DBS and the altered settings for participants in the study will be used within the indication for the treatment for advanced PD. All patients will have received the STN DBS as part of standard care. The experimental settings have already been tried during routine clinical testing of the DBS settings for optimal motor symptoms. In all other cases, minimal negative effects can be expected. Furthermore, only the activated contact will be altered, leaving the intensity, frequency and polarity unchanged. STN DBS was first performed in 1995 and has been a part of the standard care for the treatment of advanced PD in the Netherlands since then. DBS-associated problems are a decline in cognitive, mood and behavioral features, all possibly affected by the point of stimulation. In addition, two studies report an attempted suicide rate between 2 to 5 percent after STN DBS, but this was not associated with apathy. (29,30) There is no data available comparing ventral versus dorsal STN stimulation with suicide as outcome.

b. Can the primary or secondary mechanism be induced in animals and/or in *ex-vivo* human cell material?

Models for apathy in animals have been developed, but, to our knowledge, not in combination with a representative substitute for advanced PD and STN DBS. Apathy measurements cannot be performed in ex-vivo human material.

c. Selectivity of the mechanism to target tissue in animals and/or human beings

The STN DBS is highly selective for the region of stimulation, although several other neuronal regions are affected by DBS stimulation as shown in several imaging studies. (11, 30) To this day, no direct effect of STN DBS has been found on endocrinological function or other parts of the human body.

d. Analysis of potential effect

This is the first study to test the hypothesis that STN DBS related apathy can be treated with more dorsal versus ventral stimulation of the STN. The prevalence of apathy after STN DBS ranges roughly between 20% and 70% with a mean of about 50%. As of such, we are not able to provide a well-founded prediction about the potential effect. We have observed a substantial improvement of the severity of apathy in three patients in the Amsterdam UMC, location AMC. Because of these promising early results, we expect to find a lowering the severity of apathy in our sample.

e. Study population

Our study population consists of 26 patients with apathy after STN DBS for advanced PD in a multicenter double blind randomized crossover study. The fact that the subjects in our study have advanced PD confers with an older age and age-related disorders. Patients will

be told that participation in this study is no guarantee for amelioration of their symptoms, and that participation has no consequence for other forms of treatment. Patients in the control phase or not responding to the ‘study’ settings will be offered further evaluation of their symptoms by the treating neurologist. For more detailed description see Chapter 4. ‘Study Population’.

f. Interaction with other products

No interaction with other agents have been described.

g. Predictability of effect

The predictability of the effect of more dorsal stimulation of the STN on apathy and other PD symptoms is uncertain because this is the first study to test this. The SAS has been validated for apathy and is a sensitive and specific tool used in many studies in PD patients. The clinical relevance of apathy in patients receiving STN DBS is high and has a large negative impact on their quality of life. Treating this condition is of paramount importance for patients relying on DBS for the management of advanced PD.

h. Can effects be managed?

In case of discomfort or serious adverse effects, the STN DBS settings can easily be restored to the normal settings by patients or their caretakers by consulting a member of the researching team. In case of severe anxiety, benzodiazepines or other stabilizing medication or treatments are allowed. If side effects are too severe, the subject will be withdrawn from the study and considered a drop out. Participants can leave the study at any time for any reason without any consequences. The investigator can decide to withdraw a participant from the study for urgent medical reasons.

10.2 Synthesis

In conclusion, the side-effects of the experimental STN DBS settings are expected to be relatively mild and can be easily reverted by a consulting member of the research team. The most serious events that may be expected are apathetic behavior and a less than optimal motor function. Since this study will only deviate from the optimized settings by one stimulation contact, major changes in these domains are unlikely. Patients have the option to contact a member of the researching team, so in case a SAE or a SUSAR occurs, this can be quickly intercepted.

11. REFERENCES

1 Okun MS, Zeilman PR. Parkinson’s Disease: guide to deep brain stimulation therapy. National Parkinson Foundation. National Parkinson’s Foundation 2017;1:1-48.
2 Odekerken VJ, Boel JA, Schmand BA, de Haan RJ, Figee M, van den Munckhof P, Schuurman P, de Bie RMA. GPi vs STN deep brain stimulation for Parkinson disease: Three-year follow-up. Neurology 2016;86:755-61
3 The deep brain stimulation for Parkinson´s disease study group. Obeso JA, Olanow CW, Rodriguez-Oroz MC, Krack P, Kumar R, Lang AE. Deep-brain stimulation of the subthalamic nucleus or the pars interna of the globus pallidus in Parkinson's disease. N Engl J Med 2001;345:956-63
4 Witt K, Daniels C, Reiff J, Krack P, Volkmann J, Pinsker M, Krause M, Tronnier V, Kloss M, Schnitzler A et al. Neuropsychological and psychiatric changes after deep brain stimulation for Parkinson’s disease: a randomized, multicenter study. Lancet Neurology. 2008 Jul; 7: 605-14
5 Boel JA, Odekerken VJJ, Geurtsen GJ, Schmand BA, Cath DC, Figee M, van den Munckhof P, de Haan RJ, Schuurman PR, de Bie RMA. Psychiatric and social outcome after deep brain stimulation for advanced Parkinson’s disease. Movement Disorders 2015 March 31: 409-13
6 Ricciardi L, Morgante L, Epifanio A, Zibetti M, Lanotte M, Lopiano L, Morgante F. Stimulation of the subthalamic area modulating movement and behavior Parkinsonism & related disorders November 2014;20:1298–1300
7 Marin RS. Apathy: a neuropsychiatric syndrome. J Neuropsychiatry Clin Neurosci 1991;3: 243-254
8 Higuchi MA, Tsuboi Y, Inoue T, Fukuyama K, Abe H, Baba Y, Yamada T. Predictors of the emergence of apathy after bilateral stimulation of the subthalamic nucleus in patients with parkinson’s disease. Neuromodulation 2015;18:113–117
9 Martinez-Fernandez R, Pelissier P, Quesada JL, Klinger H, Lhommée E, Schmitt E, Fraix V, Chabardes S, Mertens P, Castrioto A, et al. Postoperative apathy can neutralise benefits in quality of life after subthalamic stimulation for Parkinson's disease J. neurol. Neurosurg. psychiatry 2016;87:311-8
10 Barone P, Antonini A, Colosimo C, Marconi R, Morgante L, Avarello TP, Bottacchi ECannas A, Ceravolo G, Ceravolo R, et al.The Priamo study: a multicenter assessment of nonmotor symptoms and their impact on quality of life in Parkinson’s disease. Movement Disorders. 2009;24:1641-9
11 Thobois S, Ardouin C, Lhommée E, Klinger H, Lagrange C, Xie J, Fraix V, Coelho Braga MC, Hassani R, Kistner A et al. Non-motor dopamine withdrawal syndrome after surgery for Parkinson’s disease: predictors and underlying mesolimbic denervation. Brain 2010;133:1111–1127.
12 Stefurak T, Mikulis D, Mayberg H, Lang A, Hevenor S, Pahapill P, Saint-Cyr J, Lozano A. Deep brain stimulation for Parkinson’s disease dissociates mood and motor circuits: a functional MRI case study. Movement Disorders 2003;18:1508-16
13 Czernecki V, Schüpbach M, Yaici S, Lévy R, Bardinet E, Yelnik J, Dubois B, Agid Y. Apathy Follwing Subthalamic Stimulation in Parkinson Disease: A Dopamine Responsive Symptom. Mov Dis 2008;7:964-969
14 Thobois S. Lhommee E, Klinger H, Ardouin C, Schmitt E, Bichon A, Kistner A, Castrioto A, Xie J, Fraix V, et al.. Parkinsonian apathy responds to dopaminergic stimulation of D2/D3 receptors with piribedil. Brain 2013:136;1568-1577.

15 Zoon TJ, de Bie RM, Schuurman PR, van den Munckhof P, Denys D, Figee M. Resolution of apathy after dorsal instead of ventral subthalamic deep brain stimulation for Parkinson's disease. J Neurology 2019
16 Starkstein SE Apathy in Parkinson's disease: diagnostic and etiological dilemmas. Mov Disord 2002 174-178
17 Goetz CG, Tilley BC, Shaftman SR, Stebbins GT, Fahn S, Martinez, Martin P, Poewe W, Sampaio C, Stern MB, Dodel R, et al. Movement Disorder Society-sponsored revision of the Unified Parkinson's Disease Rating Scale (MDS-UPDRS): scale presentation and clinimetric testing results. Mov Disord 200823(15):2129-70
18 Montgomery SA, Asberg M. A new depression scale designed to be sensitive to change. Britisch Journal of Psychiatry 134 (4) 382-89
19 Jenkinson C, Fitzpatrick R, Peto V, Greenhall R, Hyman N. The Parkinson's Disease Questionnaire (PDQ-39): development and validation of a Parkinson's disease summary index score. Age Ageing, 1997 Sep;26(5):353-7
20 Weintraub D, Stewart S, Shea JA, Lyons KE, Pahwa R, Driver-Dunckley ED, Adler CH, Potenza MN, Miyasaki J, Siderowf AD et al. Validation of the Questionnaire for Impulsive-compulsive disorders in Parkinson's Disease (QUIP). Mov Disord 2009 24(10): 1461-1467
21 Tomlinson CL, Stowe R, Patel S, Rick C, Gray R, Clarke CE. Systematic review of levodopa equivalency reporting in Parkinson's disease. Mov Disord 2010 25(15): 2649-53
22 Coenen VA, Rijntjes M, Prokop T, Piroth T, Amtage F, Urbach H, Reinacher PC. One-pass deep brain stimulation of dentate-rubro-thalamic tract and subthalamic nucleus for tremor-dominant or equivalent type Parkinson's disease. Acta Neurochir (2016) 158:773–781
23 Calabrese E. Diffusion tractography in deep brain stimulation surgery: a review. Front Neuroanat. 2016:2;10-45.
24 Nasreddine ZS, Philips NA, Bédirian V, Charbonneau S, Whitehead V, Collin I, Cummings JL, Chertkow H. The Montreal Cognitive Assessment, MoCA: a Brief screening tool for mild cognitive impairment. J Am Geriatr Soc 2005 Apr; 53(4)): 695-9
25 Marin RS, Biedrzycki RC, Firinciogullari S. Reliability and Validity of the Apathy Evaluation Scale. Psychiatry Research 1991 38:143-162
26 Ware J, Sherbourne C. The MOS 36-Item Short-Form Health Survey (SF-36): I. Conceptual Framework and Item Selection Medical Care 30(6):473-483, 1992
27 Leemans AJB, Sijbers J,Jones DK. ExploreDTI: a graphical toolbox for processing, analyzing, and visualizing diffusion MR data Mag Reson Med 2009, p.3537
28 Van der Linden C, Colle H. Bilateral stimulation of the subthalamic nucleus (STN) in Parkinson's disease (PD): A long-term follow-up of non-surgical, non-reversible clinical complications in 117 patients. Mov Disord 2004;19:9:S294
29 Burkhard PR, Vingerhoets FJ, Berney A, Bogousslavsky J, Villemure JG, Ghika J. Suicide after successful deep brain stimulation for movement disorders. Neurology 2004;63:2170-2
30 Le Jeune F, Drapier D, Bourguignon A, Péron J, Mesbah H, Drapier S, Sauleau P, Haegelen C, Travers D, Garin E, Malbert CH, Millet B, Vérin M. Subthalamic nucleus stimulation in Parkinson disease induces apathy. A PET study. Neurology 2009;73:1746 –1751
