# Supplementary figures and images for: A multicenter double-blind randomized crossover study comparing the impact of dorsal subthalamic nucleus deep brain stimulation versus standard care on apathy in Parkinson’s disease: a study protocol
Source: Trials. 2024 Feb 3;25:104. doi: 10.1186/s13063-024-07938-9 (PMC10837902; doi:10.1186/s13063-024-07938-9)

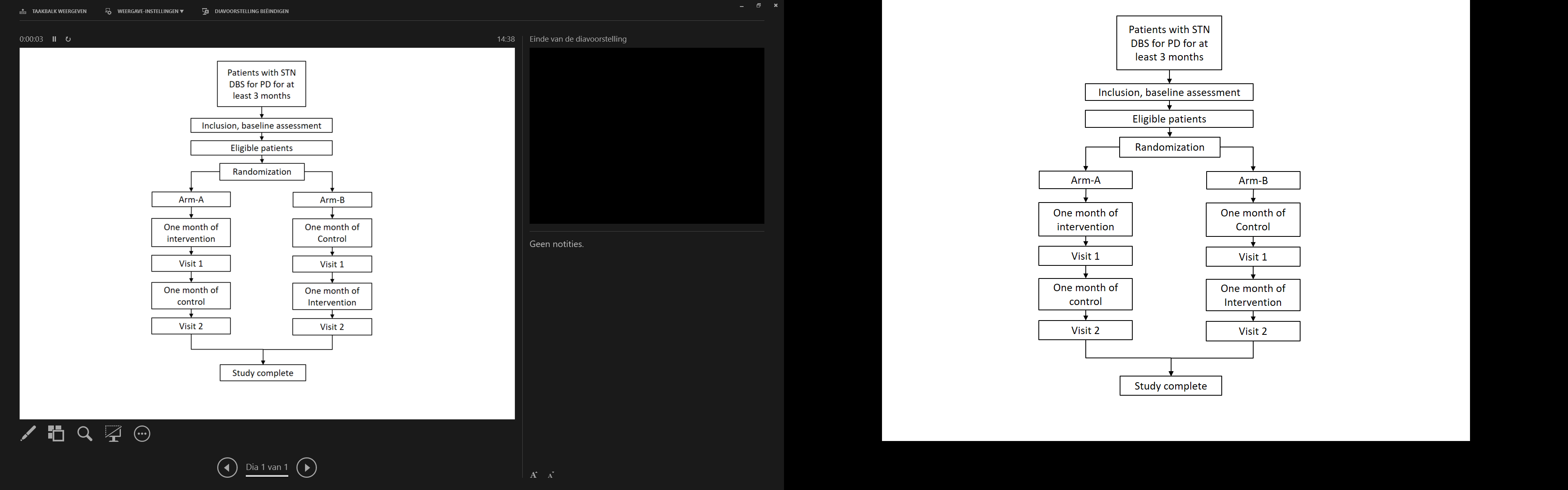


Figure 1. Inclusion and randomization

Supplement: Supplementary file 2 — Additional file 2: Figure 1. Inclusion and randomization. [file 13063_2024_7938_MOESM2_ESM.docx]
