# Supplementary material for: A multicenter double-blind randomized crossover study comparing the impact of dorsal subthalamic nucleus deep brain stimulation versus standard care on apathy in Parkinson’s disease: a study protocol
Source: Trials. 2024 Feb 3;25:104. doi: 10.1186/s13063-024-07938-9 (PMC10837902; doi:10.1186/s13063-024-07938-9)
Supplement: Supplementary file 3 — Additional file 3: Table 1. Assessment Schedule. [file 13063_2024_7938_MOESM3_ESM.docx]

|  | Pre-operative | Post-operative | Inclusion | Baseline | Visit 1 (+1 month) | Visit 2 (+2 months) | End of trial |
| --- | --- | --- | --- | --- | --- | --- | --- |
| SAS | X |  | X | X | X | X |  |
| MRI, DTI | X |  |  |  |  |  |  |
| CT-scan |  | X |  |  |  |  |  |
| In- and ex-clusioncriteria |  |  | X |  |  |  |  |
| MOCA |  |  | X |  |  |  |  |
| Baseline characteristics |  |  |  | X |  |  |  |
| MDS-UPDRS-III | X |  |  | X | X | X |  |
| PDQ-39 |  |  |  | X | X | X |  |
| QUIP | X |  |  | X | X | X |  |
| LEDD | X |  |  | X | X | X |  |
| MADRS | X |  |  | X | X | X |  |
| AES-I* |  |  |  | X | X | X |  |
| SF-36* |  |  |  | X | X | X |  |
| Suspected Arm |  |  |  |  |  |  | X |
| Preferred settings |  |  |  |  |  |  | X |

Table 1. Assessment Schedule

SAS: Starkstein’s Apathy Scale. MOCA: Montreal Cognitive Assessment. MDS-UPDRS-III: Movement Disorder Society’s Unified Parkinson’s Disease Rating Scale, motor part III. MADRS: Montgomery- Åsberg Depression Rating Scale (MADRS). PDQ-39: 39-item Parkinson’s disease Questionnaire. QUIP: Parkinson's Disease Impulsive-Compulsive Disorders Questionnaire. LEDD: levodopa-equivalent daily dosage. AES-I: Apathy Evaluation Scale, a second apathy scale rated by the informal caregiver if the patient has one. SF-36: Short-Form Health Survey. Suspected Arm: Patients will be asked to choose which arm they think they were randomized for. Preferred Settings: Patients will be asked to choose which of the settings they will continue with. *Although the timing for these questionnaires is the same as the visits for the patients, these questionnaires might be send by regular mail to the informed caregiver.
