# Supplementary material for: A multicenter double-blind randomized crossover study comparing the impact of dorsal subthalamic nucleus deep brain stimulation versus standard care on apathy in Parkinson’s disease: a study protocol
Source: Trials. 2024 Feb 3;25:104. doi: 10.1186/s13063-024-07938-9 (PMC10837902; doi:10.1186/s13063-024-07938-9)
Supplement: Supplementary file 4 — Additional file 4. World Health Trial Registration Data Set. [file 13063_2024_7938_MOESM4_ESM.docx]

1. **Primary Registry and Trial Identifying Number**
   Netherlands Trial Register (NTR), ID: NL8279.
2. **Date of Registration in Primary Registry**
   Registered on January 10^th^ 2020
3. **Secondary Identifying Numbers**
   None.
4. **Source(s) of Monetary or Material Support**
   This project is funded by the Parkinsonvereniging in the Netherlands, a institute for patients suffering from Parkinson’s Disease and their caregivers.
5. **Primary Sponsor**
   Amsterdam University Medical Centers, University of Amsterdam, Meibergdreef 9, 1105 AZ Amsterdam, The Netherlands
6. **Secondary Sponsor(s)**
   HagaZiekenhuis, Els Borst-Eilersplein 275, 2545 AA Den Haag
   Elisabeth-TweeSteden Ziekenhuis, Hilvarenbeekseweg 60, 5022 GC Tilburg
7. **Contact for Public Queries**
   Department of psychiatry, Amsterdam University Medical Centers, University of Amsterdam, Meibergdreef 9, 1105 AZ Amsterdam, The Netherlands, tel: +31 20 5913600. Email: secretariaat.psychiatrie@amsterdamumc.nl
8. **Contact for Scientific Queries**
   Coordinating investigator: Amsterdam UMC, location AMC, Department of Psychiatry. Meibergdreef 5 1100 DD Amsterdam tel: +31 20 5913600. Email: secretariaat.psychiatrie@amsterdamumc.nl
   Principal investigator: Amsterdam UMC, location AMC, department of neurology. Meibergdreef 5 1100 DD Amsterdam Tel: 020 5669111. Email: secretariaat.neurologie@amsterdamumc.nl
   Co-principal investigator: : Amsterdam UMC, location AMC, department of neurology. Meibergdreef 5 1100 DD Amsterdam Tel: 020 5669111. Email: secretariaat.psychiatrie@amsterdamumc.nl
9. **Public Title**
   Apathy and DBS in Parkinson’s disease.
10. **Scientific Title**
    A multicenter double blind randomized crossover study comparing the impact of dorsal subthalamic nucleus deep brain stimulation versus standard care on apathy in Parkinson’s disease: a study protocol.
11. **Countries of Recruitment**
    The Netherlands.
12. **Health Condition(s) or Problem(s) Studied**
    Parkinson’s Disease, apathy.
13. **Intervention(s)**
    Arm A: one month of more dorsal subthalamic nucleus stimulation (intervention) followed by one month of standard care (control).
    Arm B: one month of standard care (control) followed by one month of more dorsal subthalamic nucleus stimulation (intervention)
    Note: ‘more dorsal stimulation’ means that the most ventrally stimulating electrode will switch to 1 contact more dorsally during intervention.
14. **Key Inclusion and Exclusion Criteria**
    Inclusion: 1. Idiopathic PD 2. At least 3 months of STN DBS treatment. 3. A score of 14 or more points on the Starkstein Apathy Scale.
    Exclusion: 1. Peri-operative intracerebral complications related to STN DBS surgery. 2. Cognitive decline (Montreal Cognitive Assessment score of 25 or less. 3. Participants who are not sufficient in the Dutch language. 4. Participants who are already stimulated on the most dorsal contact point on both electrodes. 5. No informed consent.
15. **Study Type**
    - Type of study: interventional
    - Study design:
      - Method of allocation: randomized. Participants will be randomized (1:1) to arm A and B using website-based randomization in a random block design with blocks of size two and four.
      - Masking: Yes, participant and assessor. The randomization will be performed by the one of the coordinating investigator or the co-investigators in Castor, Electronic Data Capture, to conceal intervention allocation.
      - Assignment: crossover
      - Purpose: To determine the effect of more dorsal stimulation on symptoms of apathy.
16. **Date of First Enrollment**
    February 2020
17. **Sample Size**
    Total: 26
    Enrolled: 12
18. **Recruitment Status**
    Recruiting
19. **Primary Outcome(s)**
    Outcome name: Apathy
    Metric: Starkstein Apathy Scale
    Time point: one month of intervention (dorsal stimulation) and one month of control (standard care)
20. **Key Secondary Outcomes**
    Outcome name: Parkinson’s Disease motor score
    Metric: Movement Disorders Society-Unified Parkinson’s Disease Rating Scale motor part III
    Time point: one month of intervention (dorsal stimulation) and one month of control (standard care)

    Outcome name: Depression
    Metric: Montgomery- Åsberg Depression Rating Scale
    Time point: one month of intervention (dorsal stimulation) and one month of control (standard care)

    Outcome name: Parkinson’s disease related Quality of life
    Metric: 39-item Parkinson’s disease Questionnaire
    Time point: one month of intervention (dorsal stimulation) and one month of control (standard care)

    Outcome name: Impulse control disorder
    Metric: Parkinson's Disease Impulsive-Compulsive Disorders Questionnaire
    Time point: one month of intervention (dorsal stimulation) and one month of control (standard care)

    Outcome name: Antiparkinson medication
    Metric: changes in levodopa-equivalent daily dosage
    Time point: one month of intervention (dorsal stimulation) and one month of control (standard care)

    Outcome name: apathy rated by the caregiver
    Metric: Apathy Evaluation Scale
    Time point: one month of intervention (dorsal stimulation) and one month of control (standard care)

    Outcome name: burden and quality of life of the caregiver
    Metric: Short-Form Health Survey
    Time point: one month of intervention (dorsal stimulation) and one month of control (standard care)
21. **Ethics Review**
    - Status: Approved
    - Date of approval: September 23th 2019
    - Ethic committee: Medisch Ethische Toetsingscommissie (Medical Ethical Committee, METC) Amsterdam UMC, location AMC. Meibergdreef 9, 1105 AZ Amsterdam
22. **Completion date**
    Planned completion date: January 2024
23. **Summary Results**
    Not applicable
24. **IPD sharing statement**
    Plan to share IPD: Yes
    Plan description: After completion of the study and analysis of the data results will be made publically without restriction, independent of the outcome. They will be submitted for publication to an international peer‐reviewed journal.
