# Supplementary material for: A multicenter double-blind randomized crossover study comparing the impact of dorsal subthalamic nucleus deep brain stimulation versus standard care on apathy in Parkinson’s disease: a study protocol
Source: Trials. 2024 Feb 3;25:104. doi: 10.1186/s13063-024-07938-9 (PMC10837902; doi:10.1186/s13063-024-07938-9)
Supplement: Supplementary file 5 — Additional file 5. SPIRIT schedule of enrollment, interventions and assessments. [file 13063_2024_7938_MOESM5_ESM.docx]

**Schedule of enrollment:**

|  | **Enrolment** | **Allocation** | **Visits** | | **End of study** |
| --- | --- | --- | --- | --- | --- |
| **TIMEPOINT**** | ***-t_1_*** | **0** | ***t_1_*** | ***T2*** |  |
| **ENROLMENT:** |  |  |  |  |  |
| **Eligibility screen** | X |  |  |  |  |
| **Informed consent** | X |  |  |  |  |
| **Allocation** |  | X |  |  |  |
| **INTERVENTIONS:** |  |  |  |  |  |
| ***[Intervention (start Arm A) ]*** |  |  |  |  |  |
| ***[Control (start Arm B)]*** |  |  |  |  |  |
| **ASSESSMENTS:** |  |  |  |  |  |
| ***[List baseline variables]*** | X |  |  |  |  |
| ***SAS, MDS-UPDRS-III, PDQ-39, QUIP, LEDD, MADRS, AES-I, SF-36*** |  | X | X | X |  |
| ***Suspected arm, preferred settings*** |  |  |  |  | X |
