# Supplementary material for: A multicenter double-blind randomized crossover study comparing the impact of dorsal subthalamic nucleus deep brain stimulation versus standard care on apathy in Parkinson’s disease: a study protocol
Source: Trials. 2024 Feb 3;25:104. doi: 10.1186/s13063-024-07938-9 (PMC10837902; doi:10.1186/s13063-024-07938-9)
Supplement: Supplementary file 8 — Additional file 8. Model consent form (Dutch). [file 13063_2024_7938_MOESM8_ESM.pdf]

Bijlage D: toestemmingsformulier proefpersoon

Behandeling van DBS-gerelateerde apathie

- Ik heb de informatiebrief gelezen. Ook kon ik vragen stellen. Mijn vragen zijn voldoende beantwoord. Ik had genoeg tijd om te beslissen of ik meedoe.
- Ik weet dat meedoen vrijwillig is. Ook weet ik dat ik op ieder moment kan beslissen om toch niet mee te doen of te stoppen met het onderzoek. Daarvoor hoef ik geen reden te geven.
- Ik geef toestemming voor het informeren van mijn huisarts en specialist dat ik meedoe aan dit onderzoek.
- Ik weet dat sommige mensen mijn gegevens kunnen inzien. Die mensen staan vermeld in deze informatiebrief.
- Ik geef toestemming voor het verzamelen en gebruiken van mijn gegevens op de manier en voor de doelen die in de informatiebrief staan.
- Ik geef toestemming om mijn gegevens op de onderzoekslocatie nog [15] jaar na dit onderzoek te bewaren.
- Ik geef □ **wel**
  □ **geen** toestemming om mij na dit onderzoek opnieuw te benaderen voor een vervolgonderzoek.
- Ik wil □ **wel**
  □ **niet** geïnformeerd worden over welke behandeling ik heb gehad/in welke groep ik zat.
- Ik wil meedoen aan dit onderzoek.

Naam proefpersoon:
Handtekening: Datum : __ / __ / __

Ik verklaar dat ik deze proefpersoon volledig heb geïnformeerd over het genoemde onderzoek.

Als er tijdens het onderzoek informatie bekend wordt die de toestemming van de proefpersoon zou kunnen beïnvloeden, dan breng ik hem/haar daarvan tijdig op de hoogte.

Naam onderzoeker (of diens vertegenwoordiger):
Handtekening: Datum: __ / __ / __

De proefpersoon krijgt een volledige informatiebrief mee, samen met een kopie van het getekende toestemmingsformulier.
